# Serum thyroid-stimulating hormone levels are not associated with exercise capacity and lung function parameters in two population-based studies

**DOI:** 10.1186/1471-2466-14-145

**Published:** 2014-09-02

**Authors:** Till Ittermann, Sven Gläser, Ralf Ewert, Stephan Felix, Henry Völzke, Marcus Dörr

**Affiliations:** 1 Institute for Community Medicine, Ernst Moritz Arndt University, Walther Rathenau Str. 48, D-17475 Greifswald, Germany; 2 Department of Internal Medicine B – Cardiology, Intensive Care, Pulmonary Medicine and Infectious Diseases, University Medicine Greifswald, Greifswald, Germany; 3 DZHK (German Center for Cardiovascular Research), partner site Greifswald, Greifswald, Germany

**Keywords:** TSH, Hyperthyroidism, Hypothyroidism, Exercise capacity, Lung function, Oxygen uptake, Spirometry, Population-based study, Epidemiology

## Abstract

**Background:**

Thyroid dysfunction has been described to be linked to a variety of cardiovascular morbidities. Through this pathway thyroid function might also be associated with cardiorespiratory function and exercise capacity. So far only few patient-studies with small study populations investigated the association between thyroid dysfunction and exercise capacity. Thus, the aim of our study was to investigate the association of serum thyroid-stimulating hormone (TSH) levels with lung function and cardiopulmonary exercise testing (CPET) in the general population.

**Methods:**

Data from the two independent cross-sectional population-based studies (Study of Health in Pomerania [SHIP] and SHIP-Trend-0) were pooled. SHIP was conducted between 2002 and 2006 and SHIP-Trend-0 between 2008 and 2012. Participants were randomly selected from population registries. In total, 4206 individuals with complete data were available for the present analysis. Thyroid function was defined based on serum TSH levels. Lung function was evaluated by forced expiratory volume in 1 s and forced vital capacity. CPET was based on symptom limited exercise tests on a bicycle in a sitting position according to a modified Jones protocol. Associations of serum TSH levels with lung function and CPET parameters were analysed by multivariable quantile regression adjusted for age, sex, height, weight, use of beta blockers, smoking status, and physical activity.

**Results:**

Serum TSH levels, used as continuously distributed variable and categorized according to the clinical cut-offs 0.3 and 3.0 mIU/L or according to quintiles, were not consistently associated with parameters of lung function or CPET.

**Conclusions:**

Our results suggest that thyroid dysfunction is not associated with lung function and cardiopulmonary exercise capacity in the general population.

## Background

Thyroid dysfunction has been described to be linked to a variety of cardiovascular morbidities [1]. For hyperthyroidism associations with atrial fibrillation [2], left ventricular hypertrophy [3], carotid atherosclerosis [4,5], and high fibrinogen levels [6] have been reported. On the other hand hypothyroidism is associated with altered lipid metabolism [7,8], endothelial dysfunction [9], and an overall increased atherosclerotic risk [10]. The association between thyroid dysfunction and cardiovascular diseases might in part be linked with or paralleled by reduced lung function which is strongly associated with increased cardiovascular morbidity and mortality [11,12]. In fact, evidence for such an association exists. Thus, thyroid dysfunctions are for example associated with pulmonary hypertension [13]. It has also been demonstrated that hyperthyroidism is accompanied by a reduced muscle strength resulting in impaired ventilation under exercise [14,15]. Furthermore, it has been demonstrated that the cardiovascular capacity is reduced in hyperthyroid patients, which might be related to a disturbed chronotropic regulation [16] and can be restored by treatment with antithyroid medication [17]. Likewise, also hypothyroid states have been shown to be related to lung function impairment. A link between hypothyroidism and decreased lung function might be explained by alveolar hypoventilation, decreased inspiratory muscle strength, and decreased respiratory rates [18,19]. In hypothyroid patients it has been shown that treatment with thyroxine results in an improved exercise cardiopulmonary reserve [20].

So far several patient-studies with small study populations investigated the association of hypo- and hyperthyroidism with lung function [18,21-26] and exercise capacity [23,27-30]. While most of the patient studies demonstrated significant associations between thyroid dysfunction and lung function [18,22-24,26], results from studies investigating the impact of hyperthyroidism on exercise capacity are inconsistent [23,30]. Kahaly et al. [23] showed a reduced exercise capacity in patients with untreated overt hyperthyroidism, whereas Portella and coworkers did not demonstrate an association between hyperthyroidism and exercise capacity in female patients with subclinical hyperthyroidism [30]. Regarding hypothyroidism, one study [27] showed an impaired exercise capacities in patients under long-term levothyroxine treatment for nontoxic goiter or differentiated thyroid carcinoma in comparison to healthy controls. In agreement with this, another study [28] demonstrated reduced exercise capacities in patients with exogenous subclinical hypothyroidism compared to controls, but one-year-change of exercise capacity did not differ among groups of levothyroxine treatment and placebo groups [28]. In line with this, another study reported an association between low free triiodothyronine levels and impaired exercise capacity in patients with heart failure [29].

The discrepancy in results among previous studies investigating associations between thyroid dysfunction and exercise capacity is probably referred to the fact that all those studies were conducted in selected patient populations. Furthermore, it is unclear whether the significant findings between thyroid dysfunction and lung function detected in the patient studies can be extended to the general population, because potential associations of thyroid dysfunction with lung function and exercise capacity has never been analysed in population-based samples. Against this background we aimed to investigate the association of thyroid function state as determined by serum thyroid-stimulating hormone (TSH) levels with parameters of lung function and cardiopulmonary exercise testing (CPET) in two independent population-based studies conducted in Northeast Germany.

## Methods

### Study population

Analyses are based on data from two independent cohorts of the Study of Health in Pomerania (SHIP), conducted in Northeast Germany [31]. In the first SHIP cohort (SHIP-0) 6267 eligible subjects were randomly selected from population registries. Of those, 4308 individuals were examined between 1997 and 2001 (68.8%). Between 2002 and 2006 all participants were re-invited for an examination follow-up (SHIP-1), in which 3300 subjects aged 25 to 85 years took part (1589 men and 1711 women; 83.5% of all eligible subjects). Of those, 1708 individuals volunteered for CPET and spirometry (52.0%).

For the second SHIP cohort (SHIP-Trend-0) 8800 eligible subjects were randomly selected from population registries and 4422 individuals aged 20 to 81 years participated (response 50.3%) between 2008 and 2012. In SHIP-Trend-0 in total 2678 individuals volunteered CPET and spirometry examinations (60.6%). All participants gave informed written consent and both studies followed the recommendations of the Declaration of Helsinki and were approved by the Ethics Committee of the University of Greifswald.

From SHIP-1 and SHIP-Trend-0, we excluded 34 and 146 participants, respectively, with missing data in any of the considered variables. Furthermore, we excluded 22 individuals in SHIP-Trend-0, who weighed more than 120 kg and therefore had to use a recumbent bike for spiroergometry. Measurements from the recumbent bike are not comparable with results from the standard cycle ergometer. In total, our analyses are based on a study population consisting of 4184 individuals (2110 women).

### Assessments

Physical activity and smoking status were assessed by computer-assisted personal interviews. Subjects who participated in physical training during summer or winter for at least one hour a week were classified as being physically active. Smokers were categorized into three categories (lifetime non-smokers, former smokers, and current smokers). Beta blocker use was defined by the anatomic-therapeutical-chemical (ATC) code C07. Height and weight were measured for calculation of the body mass index (BMI).

Blood samples were taken non-fasting in SHIP-1. In SHIP-Trend-0 75% of the blood samples were taken fasting. Serum TSH levels were measured by immunochemiluminiscent procedures (SHIP-1: Immulite 2000, Third Generation, DPC, Los Angeles, USA; SHIP-Trend-0: Vista, Siemens, Eschborn, Germany). In SHIP-Trend-0 median serum TSH levels were comparable between fasting and non-fasting individuals (1.15 mIU/L vs 1.10 mIU/L). Low and high serum TSH levels were defined by the cut-offs 0.3 mIU/L and 3.0 mIU/L according to Baskin et al. [32]. Furthermore, individuals were classified into five groups according to TSH quintiles.

In both studies, CPET was performed using the same calibrated electromagnetically braked cycle ergometer (Ergoselect 100, Ergoline, Germany) with a physician in attendance according to a modified Jones protocol: 3 min of rest, 1 min of unloaded cycling at 60 rpm, stepwise increases in work load of 16 W/min until symptom-limited or terminated by the physician due to chest pain or ECG abnormalities, and 5 min of recovery [33]. From spirometry we used forced expiratory volume in 1 s (FEV_1_), forced vital capacity (FVC), and its ratio (FEV_1_/FVC) to characterize resting lung function [34]. Furthermore, we used the following parameters from CPET: peak oxygen uptake (peakVO_2_), oxygen uptake at anaerobic threshold (VO_2_@AT), slope of the efficiency of ventilation in removing carbon dioxide (V_E_ vs. VCO_2_ slope), oxygen pulse (O_2_HR), maximum power, and exercise duration [35].

### Statistical analyses

Continuous variables are expressed as median, 25th, and 75th percentile; categorical variables as absolute numbers and percentages. Serum TSH levels were associated with parameters of CPET and spirometry by median regression adjusted for age, sex, height, weight, beta blocker use, smoking status, physical activity, study (SHIP-1 or SHIP-Trend-0), and time between core examination and CPET examination. Median regression was used, because for some outcomes the residuals were not normally distributed. Participants significantly differed from non-participants of the CPET examination regarding serum TSH levels, age, body mass index, beta blocker use, physical activity, current smoking, and education [36]. Particularly, participating individuals were younger, had a lower BMI, and were less often smokers and physically inactive than non-participants. To account for these differences we applied inverse probability weights. This procedure gives individuals from groups, which are more likely to drop out, a stronger weight in the analyses compared to individuals from groups with less drop outs. To account for possible non-linear relationships between serum TSH levels or any of the confounders with the respective outcome multivariable fractional polynomials were tested [37]. A p < 0.05 was considered as statistically significant. All analyses were carried out using Stata 12.1 (Stata Corporation, College Station, TX, USA).

## Results

Serum TSH levels were higher in SHIP-Trend-0 than in SHIP-1 (1.14 mIU/L vs. 0.78 mIU/L). Measurements of CPET were comparable among the two studies, but FEV_1_/FVC was 5.5% lower in SHIP-Trend-0 than in SHIP-1. In the pooled population most measurements of spirometry (FEV_1_ and FVC) and CPET (peakVO_2_, VO_2_@AT, O_2_HR, maximum power, and exercise duration) were lower in individuals with low TSH compared to individuals with serum TSH levels in the reference range, while there were substantial differences between individuals with high TSH and individuals with TSH in the reference range only for VO_2_@AT and O_2_HR (Table 1).

**Table 1:** Characteristics of the pooled study population stratified by serum TSH levels

	**0.3 < TSH ≤ 3.0 mIU/L**	**TSH ≤ 0.3 mIU/L**	**TSH > 3.0 mIU/L**
	**(n = 3877)**	**(n = 200)**	**(n = 107)**
**Age; years**	52 (41; 63)	57 (47; 68)	48 (37; 58)
**Males**	1949 (50.3%)	85 (42.5%)	40 (37.4%)
**BMI; kg/m**^**2**^	27.3 (24.5; 30.6)	27.7 (24.3; 31.0)	26.9 (23.6; 30.0)
**Physical activity**	1986 (51.2%)	86 (43.0%)	61 (57.0%)
**Former smokers**	1395 (36.0%)	91 (45.5%)	35 (32.7%)
**Current smokers**	861 (22.2%)	33 (16.5%)	23 (21.5%)
**Systolic blood pressure, mmHg**	128 (116; 140)	127 (114; 140)	124 (113; 137)
**Diastolic blood pressure, mmHg**	78 (72; 85)	79 (72; 85)	78 (72; 85)
**Beta blocker use**	862 (22.2%)	58 (29.0%)	22 (20.6%)
**Thyroid medication use**	363 (9.4%)	59 (29.5%)	24 (22.4%)
**Education**			
- **< 10 years**	900 (23.2%)	69 (34.5%)	12 (11.2%)
- **= 10 years**	1947 (50.2%)	96 (48.0%)	57 (53.3%)
- **> 10 years**	1029 (26.6%)	35 (17.5%)	38 (37.5%)
**FEV**_**1**_**; l**	3.2 (2.6; 3.8)	3.0 (2.5; 3.6)	3.3 (2.7; 3.8)
**FVC; l**	4.0 (3.3; 4.8)	3.6 (3.0; 4.4)	4.0 (3.3; 4.7)
**FEV**_**1**_**/FVC; %**	81.8 (77.5; 85.8)	82.6 (78.4; 86.6)	81.4 (77.8; 85.3)
**peakVO**_**2**_**; ml/min**	1900 (1500; 2400)	1703 (1450; 2092)	1905 (1507; 2450)
**VO**_**2**_**@AT; ml/min**	1000 (850; 1200)	975 (800; 1175)	950 (850; 1200)
**O**_**2**_**HR; ml/beat**	12.8 (10.4; 15.6)	12.0 (10.5; 15.0)	12.4 (9.5; 14.9)
**V**_**E **_**vs. VCO**_**2 **_**slope**	26 (24; 29)	26 (24; 29)	26 (24; 29)
**Maximum power; watt**	148 (116; 196)	132 (116; 164)	148 (116; 180)
**Exercise duration; minutes**	9.1 (7.2; 11.4)	8.1 (6.4; 10.1)	9.1 (7.2; 11.1)

Median, 25th, and 75th percentile (continuous variables); absolute numbers and percentages (categorical variables).

### Serum TSH levels and lung function

Serum TSH levels over the full range were significantly associated with FEV_1_/FVC in a linear fashion, while there was no association to FEV_1_ and FVC values (Table 2). In comparison to serum TSH levels within the reference range, FEV_1_, FVC, and FEV_1_/FVC values did neither differ for low nor for high serum TSH levels. Serum TSH levels within the reference range were significantly associated with FEV_1_/FVC, but not with FEV_1_ and FVC values. For FEV_1_, FVC, and FEV_1_/FVC there were no significant differences between the third and the other TSH quintiles (Table 3).

**Table 2:** Association between TSH and parameters of spirometry and cardiopulmonary exercise testing

	**Model 1**	**Model 2**		**Model 3**
	**TSH full range**	**TSH < 0.3**^**#**^	**TSH > =3**^**#**^	**TSH in the reference range**
	**β (95%-CI)**	**β (95%-CI)**	**β (95%-CI)**	**β (95%-CI)**
**FEV**_**1**_**; l**	-0.01 (-0.04; 0.01)	0.06 (-0.06; 0.17)	-0.06 (-0.14; 0.02)	0.01 (-0.03; 0.04)
**FVC; l**	-0.003 (-0.034; 0.028)	0.03 (-0.10; 0.17)	-0.08 (-0.21; 0.04)	0.02 (-0.03; 0.06)
**FEV**_**1**_**/FVC; %**	-0.35* (-0.65; -0.06)	-0.06 (-1.23; 1.12)	-0.78 (-2.02; 0.47)	-0.48* (-0.91; -0.04)
**peakVO**_**2**_**; ml/min**	1.2 (-18.1; 20.4)	19.2 (-46.4; 84.8)	3.0 (-98.5; 104.5)	12.9 (-15.4; 41.3)
**VO**_**2**_**@AT; ml/min**	-4.7 (-14.8; 5.5)	14.0 (-19.3; 47.4)	-9.3 (-52.0; 33.5)	-4.2 (-18.2; 9.9)
**O**_**2**_**HR; ml/beat**	-0.11* (-0.22; -0.01)	0.33 (-0.14; 0.81)	-0.31 (-0.88; 0.26)	-0.08 (-0.25; 0.08)
**V**_**E **_**vs. VCO**_**2 **_**slope**	0.11 (-0.06; 0.29)	-0.44 (-1.08; 0.19)	0.05 (-0.69; 0.79)	0.21 (-0.05; 0.46)
**Maximum power; watt**	1.3 (-0.1; 2.7)	0.1 (-5.7; 6.0)	-0.4 (-11.1; 10.3)	1.9 (-0.1; 3.9)
**Exercise duration; minutes**	0.02 (-0.08; 0.12)	0.02 (-0.33; 0.37)	-0.16 (-0.62; 0.29)	0.07 (-0.07; 0.20)

Median regression adjusted for age, sex, body mass index, beta blocker intake, smoking status, physical activity, study and time between examinations; *p < 0.05; ^#^Reference: TSH in the reference range.

**Table 3:** Association between quintiles of TSH and parameters of spirometry and cardiopulmonary exercise testing

	**First quintile**^**#**^	**Second quintile**^**#**^	**Fourth quintile**^**#**^	**Fifth quintile**^**#**^
	**(0 – 0.5745 mIU/L)**	**(0.5745 – 0.839 mIU/L)**	**(1.14 – 1.59 mIU/L)**	**(1.59 – 10.00 mIU/L)**
	**β (95%-CI)**	**β (95%-CI)**	**β (95%-CI)**	**β (95%-CI)**
**FEV**_**1**_**; l**	0.00 (-0.06; 0.07)	0.02 (-0.05; 0.08)	-0.01 (-0.07; 0.05)	0.01 (-0.05; 0.07)
**FVC; l**	-0.01 (-0.08; 0.06)	-0.01 (-0.08; 0.07)	-0.002 (-0.073; 0.069)	0.005 (-0.073; 0.083)
**FEV**_**1**_**/FVC; %**	0.31 (-0.38; 0.99)	-0.02 (-0.75; 0.71)	-0.14 (-0.80; 0.52)	-0.45 (-1.13; 0.24)
**peakVO**_**2**_**; ml/min**	-32.3 (-73.1; 8.5)	-48.5* (-89.2: -7.9)	-35.3 (-79.8; 9.3)	-16.6 (-60.5; 27.4)
**VO**_**2**_**@AT; ml/min**	2.4 (-21.8; 26.6)	-3.7 (-28.3; 20.9)	-11.1 (-34.5; 12.4)	-8.2 (-30.7; 14.3)
**O**_**2**_**HR; ml/beat**	-0.06 (-0.35; 0.24)	-0.25 (-0.53; 0.02)	-0.28 (-0.56; 0.01)	-0.32* (-0.59; -0.05)
**V**_**E **_**vs. VCO**_**2 **_**slope**	-0.20 (0.66; 0.27)	-0.24 (-0.71; 0.23)	0.20 (-0.23; 0.64)	0.04 (-0.40; 0.48)
**Maximum power; watt**	-3.7* (-7.1; -0.3)	-3.1 (-6.7; 0.5)	-2.7 (-6.0; 0.6)	0.2 (-3.2; 3.5)
**Exercise duration; minutes**	-0.22* (-0.41; -0.03)	-0.15 (-0.35; 0.04)	-0.18 (-0.39; 0.03)	-0.07 (-0.29; 0.15)

Median regression adjusted for age, sex, height, weight, beta blocker intake, smoking status, physical activity, and time between examinations.

*p < 0.05.

^#^Reference: Third quintile 0.839 – 1.14 mIU/L.

### Serum TSH levels and CPET

Serum TSH levels over the full range were inversely associated with O_2_/HR in multivariable linear regression, but there was no association with peakVO_2_, VO_2_@AT, V_E_ vs. VCO_2_ slope, maximum power, and exercise duration (Table 2). Compared to serum TSH levels within the reference range, CPET parameters did neither significantly differ for low nor for high serum TSH levels. Likewise, serum TSH levels within the reference range were not associated with any of the CPET parameters. Regression analyses with quintiles of serum TSH levels revealed in total four significant associations to CPET parameters (Table 3). PeakVO_2_ values were significantly lower in the 2nd TSH quintile in comparison to the 3rd TSH quintile, but peakVo_2_ values did not significantly differ between the 1st and the 3rd TSH quintile. Maximum power and exercise duration values were significantly lower in the 1st TSH quintile than in the 3rd TSH quintile. O_2_/HR values were significantly lower in the 5th than in the 3rd TSH quintile.

For sensitivity analysis we excluded all individuals treated with thyroid medication. In this subpopulation regression results did not differ significantly from those in the total population (Additional file 1: Table S1).

## Discussion

In data pooled from two independent large population-based studies from Northeast-Germany we detected no consistent associations between thyroid function state as measured by serum TSH levels and lung function or exercise capacity. The statistically significant results that were found in some sub-analyses of our study might be referred to the problem of multiple testing. The likelihood to detect a significant association increases with the number of tests performed. If we assume the Bonferroni corrected p-value threshold of 0.0007, none of the tests performed in our analysis would be statistically significant. Furthermore, the detected significant associations at the 0.05 α-level do not give a uniform picture. For example, FEV_1_/FVC was related to serum TSH levels over the full range and serum TSH levels within the reference range, but not with TSH quintiles or hypo- and hyperthyroidism. Likewise, inconsistent findings were detected for peakVO_2_, O_2_/HR, and maximum power. Thus, our results suggest that there is no evidence of an association between thyroid dysfunction and lung function and CPET in the general population.

### Thyroid dysfunction and lung function

There are several small patient studies that investigated putative associations between hypo- or hyperthyroidism with spirometry parameters [18,21-26]. In two of these studies patients with hypothyroidism had lower FEV-1 and FVC values than euthyroid controls [24,26], while in two other patient studies such associations were not detected [21,25]. Differences between these patient studies might be related to the low number of included individuals and different medical conditions of individuals in those studies. The studies, which did not detect an association between hypothyroidism and lung function, consist of individuals with COPD [25] or of individuals with treated hypothyroidism [21], while the studies, which detected significant associations, included individuals with untreated hypothyroidism [24,26]. On the other hand, patient studies consistently reported a lower lung function in hyperthyroid patients than in euthyroid controls [22,23,26]. Thus, the findings of the patient studies hypothesize that there might also be an association between thyroid dysfunction, especially hyperthyroidism, and lung function. In our study, however, we did not detect a significant association between serum TSH levels and lung function based on a comprehensive set of spirometry parameters. An explanation for the lack of a significant finding might be that the number of individuals with clinically relevant thyroid dysfunction might be relatively low as can be seen by the relatively low proportion of thyroid medication intake in individuals with serum TSH levels outside the reference range. Even though we have no serum free triiodothyronine (fT3) or thyroxine (fT4) levels measured in SHIP-1 and SHIP-Trend-0 to distinct overt and subclinical forms of thyroid dysfunction, findings from SHIP-0, the baseline of SHIP-1, indicate that the prevalence of overt thyroid dysfunctions might be low in our study population. Thus, in SHIP-0 there were only 28 individuals (0.7%) with overt hyperthyroidism and 36 individuals (0.9%) with over hypothyroidism. Hence, the results of our study might argue that lung function is not reduced due to mild forms of hypo- or hyperthyroidism.

### Thyroid dysfunction and CPET

With respect to the potential association between hyperthyroid dysfunction and CPET, the findings of our study are in agreement with a small study [30], in which exercise tolerance of 14 females with subclinical hyperthyroidism did not differ from 15 age-matched apparently healthy controls. In contrast, another study demonstrated a reduced exercise capacity in 42 patients with untreated hyperthyroidism that was significantly increased 6 months later when euthyroidism was restored by methimazole [23]. Similarly, another study [38] reported a reduced exercise capacity in 15 hyperthyroid patients. Exercise testing was repeated in nine of these subjects after treatment with carbimazole, showing markedly improved exercise parameters [38]. The discrepancy between our study and the results of the other two studies [23,38] might be referred mainly to the different study populations. Those studies [23,38] analysed data of patients with subclinical or overt hyperthyroidism and/or the effects of restoration of euthyroidism. In contrast, our analyses are based on a population based sample which is characterized by fewer subjects with clinically relevant hyperthyroid states, which might explain the lack of a significant finding in our analyses.

With respect to hypothyroidism, two studies are available that, in contrast to our study, found consistent associations with exercise capacity. Thus, one study demonstrated lowered exercise capacity in hypothyroid patients under long-time levothyroxine treatment [27]. Another study showed a reduced exercise tolerance in hypothyroid patients with Hashimoto thyroiditis in comparison to matched controls at baseline but no changes in these patients after one year use of levothyroxine [28]. The results of those studies [27,28] might argue for an impaired exercise capacity only in individuals with overt states of hypothyroidism and/or long-time thyroid hormone treatment.

### Strength and limitations

Strengths of our study are its population-based design, which allows generalization for the background population of Northeast Germany, and the large number of individuals included. A limitation is that we were not able to divide hypo- and hyperthyroidism into its overt and subclinical forms due to the lack of free triiodothyronine and free thyroxine levels. However, results from the baseline SHIP examinations, for which these measurements were available, indicate that most of the participants with thyroid dysfunction have the subclinical form [39]. Furthermore, there were relevant differences between participants and non-participants of the CPET examinations. We account for these differences in our analysis by applying inverse probability weights.

## Conclusions

Our results suggest that thyroid dysfunction is not associated with lung function and CPET in the general population.

## Competing interests

The authors declare that they have no competing interests.

## Authors’ contributions

TI carried out the statistical analyses and drafted the manuscript. SG, RE, SF and MD participated in the coordination of the study and drafted the manuscript. HV participated in the design of the study and drafted the manuscript. All authors read and approved the final manuscript.

## Pre-publication history

The pre-publication history for this paper can be accessed here:

http://www.biomedcentral.com/1471-2466/14/145/prepub

## Supplementary Material

Additional file 1: Table S1 Association between TSH and parameters of spirometry and cardiopulmonary exercise testing in individuals without thyroid medication intake. Click here for file
