# Supplementary material for: Serum thyroid-stimulating hormone levels are not associated with exercise capacity and lung function parameters in two population-based studies
Source: BMC Pulm Med. 2014 Sep 2;14:145. doi: 10.1186/1471-2466-14-145 (PMC4236747; doi:10.1186/1471-2466-14-145)
Supplement: Additional file 1: Table S1 — Association between TSH and parameters of spirometry and cardiopulmonary exercise testing in individuals without thyroid medication intake. [file 1471-2466-14-145-S1.docx]

Additional file 1: Association between TSH and parameters of spirometry and cardiopulmonary exercise testing in individuals without thyroid medication intake.

|  | **Model 1** | **Model 2** | | **Model 3** |
| --- | --- | --- | --- | --- |
|  | **TSH full range**  **β (95%-CI)** | **TSH < 0.3^#^**  **β (95%-CI)** | **TSH>= 3^#^**  **β (95%-CI)** | **TSH in the reference range**  **β (95%-CI)** |
| **FEV_1_; l** | -0.01 (-0.04; 0.02) | 0.00 (-0.14; 0.14) | -0.05 (-0.15; 0.05) | 0.01 (-0.05; 0.03) |
| **FVC; l** | -0.007 (-0.041; 0.027) | -0.02 (-0.16; 0.12) | -0.07 (-0.22; 0.07) | 0.02 (-0.04; 0.07) |
| **FEV_1_/FVC; %** | -0.42* (-0.76; -0.09) | -1.02 (-2.38; 0.35) | -1.28 (-2.77; 0.22) | -0.37 (-0.85; 0.11) |
| **peakVO_2_; ml/min** | 2.2 (-23.8; 28.1) | 21.3 (-68.7; 111.3) | -3.7 (-117.4; 111.3) | 12.0 (-21.0; 45.0) |
| **VO_2_@AT; ml/min** | -4.0 (-16.0; 8.1) | 13.0 (-30.6; 56.7) | -3.9 (-56.3; 48.5) | -2.7 (-19.0; 13.6) |
| **O_2_HR; ml/beat** | -0.11 (-0.25; 0.03) | 0.32 (-0.33; 0.96) | -0.23 (-0.80; 0.33) | -0.08 (-0.28; 0.11) |
| **V_E_ vs. VCO_2_ slope** | 0.11 (-0.07; 0.29) | -0.61 (-1.34; 0.12) | 0.12 (-0.59; 0.82) | 0.18 (-0.11; 0.48) |
| **Maximum power; watt** | 1.2 (-0.6; 3.0) | 0.0 (-5.9; 5.9) | 1.8 (-9.9; 13.5) | 2.0 (-0.2; 4.3) |
| **Exercise duration; minutes** | 0.03 (-0.08; 0.13) | 0.06 (-0.35; 0.47) | -0.10 (-0.60; 0.40) | 0.09 (-0.05; 0.24) |

Median regression adjusted for age, sex, body mass index, beta blocker intake, smoking status, physical activity, study and time between examinations; *p<0.05; # Reference: TSH in the reference range
